# Description of long-term monitoring of farmland biodiversity in a LTSER

**DOI:** 10.1016/j.dib.2018.05.028

**Published:** 2018-05-19

**Authors:** Vincent Bretagnolle, Elsa Berthet, Nicolas Gross, Bertrand Gauffre, Christine Plumejeaud, Sylvie Houte, Isabelle Badenhausser, Karine Monceau, Fabrice Allier, Pascal Monestiez, Sabrina Gaba

**Affiliations:** a CEBC, UMR 7372, CNRS & Université de la Rochelle, 79360 Villiers-en-Bois , France; b LTSER “Zone Atelier Plaine & Val de Sèvre”, CNRS, 79360 Villiers-en-Bois , France; c UMR SADAPT, INRA, AgroParisTech, Université Paris-Saclay, 16 rue Claude Bernard, 75005 Paris, France; d UMR LIENSs 7266 Université de la Rochelle et CNRS, 2 rue Olympe de Gouges, 17000 La Rochelle, France; e USC 1339, Centre d’Etudes Biologiques de Chizé, INRA, Villiers en Bois, 79360 Beauvoir sur Niort, France; f ITSAP-Institut de l’Abeille, Domaine Saint-Paul, CS 40509, 84914 Avignon, France; g UMT PrADE, CS 40509, 84914 Avignon, France; h BioSP, INRA, 84914 Avignon, France; i Agroécologie, AgroSup, INRA, Université de Bourgogne, 21065 Dijon, France

## Abstract

Understanding the response of biodiversity to management, land use and climate change is a major challenge in farmland to halt the decline of biodiversity. Farmlands shelter a wide variety of taxa, which vary in their life cycle and habitat niches. Consequently, monitoring biodiversity from sessile annual plants to migratory birds requires dedicated protocols. In this article, we describe the protocols applied in a long-term research platform, the LTSER Zone Atelier “Plaine & Val de Sèvre” (for a full description see Bretagnolle et al. (2018) [1]). We present the data in the form of the description of monitoring protocols, which has evolved through time for arable weeds, grassland plants, ground beetles, spiders, grasshoppers, wild bees, hoverflies, butterflies, small mammals, and farmland birds (passerines, owls and various flagship species).

**Specifications Table**

**Table:** 

Subject area	*Biodiversity conservation, Ecology, Population and Community dynamic*
More specific subject area	*Analysing long-term trends of biodiversity in farmland*
Type of data	Text file
How data was acquired	Sampling and survey
Data format	Raw
Experimental factors	
Experimental features	
Data source location	LTSER Zone Atelier “Plaine & Val de Sèvre”, South of the Deux Sèvres Department, France
Data accessibility	In Supplementary Material with this article
Related research article	Bretagnolle et al. 2018. Biodiversity, ecosystem services and citizen science: the value of long term monitoring in farmland landscapes for sustainable agriculture. *Science of The Total Environment* 627: 822-834

**Value of the Data**

• We developed protocols to monitor changes in the main taxonomic groups of farmland biodiversity at a large number of sampling points (up to 2000 for some taxa) on a regional level.
• We based the protocols on standardized methods from the literature and adapted them based on up to 25-year experience with implementation in the field.
• We provide detailed descriptions of the protocols so that these can be replicated by others projects involving long-term monitoring of biodiversity in agricultural landscapes.
• We encourage others projects to adopt these standardized methods in order to harmonize monitoring, and thus increase the comparative value of the results across long-term research sites.

## 1 Data

The data described in this article present the protocols for a spatially explicit monitoring of aboveground biodiversity. These protocols are applied in a long-term research platform, the LTSER Zone Atelier “Plaine & Val de Sèvre” (hereafter ZA PVS; for a full description see Bretagnolle et al. [1]). The data comprise information on the spatial monitoring protocols of arable weeds, grassland plants, ground beetles, spiders, grasshoppers, wild bees, hoverfly, butterfly, small mammals, and farmland birds (passerine and nocturnal birds and flagship species). For each taxon, we deliver the type and size of the sampling unit, the number of sampling units and the timing of sampling (Table 1). Details on the spatial monitoring strategy for each taxon are provided in Supplementary material. Biodiversity data are available upon request to the corresponding author.

## 2 Monitoring design

**Fig. 1:**
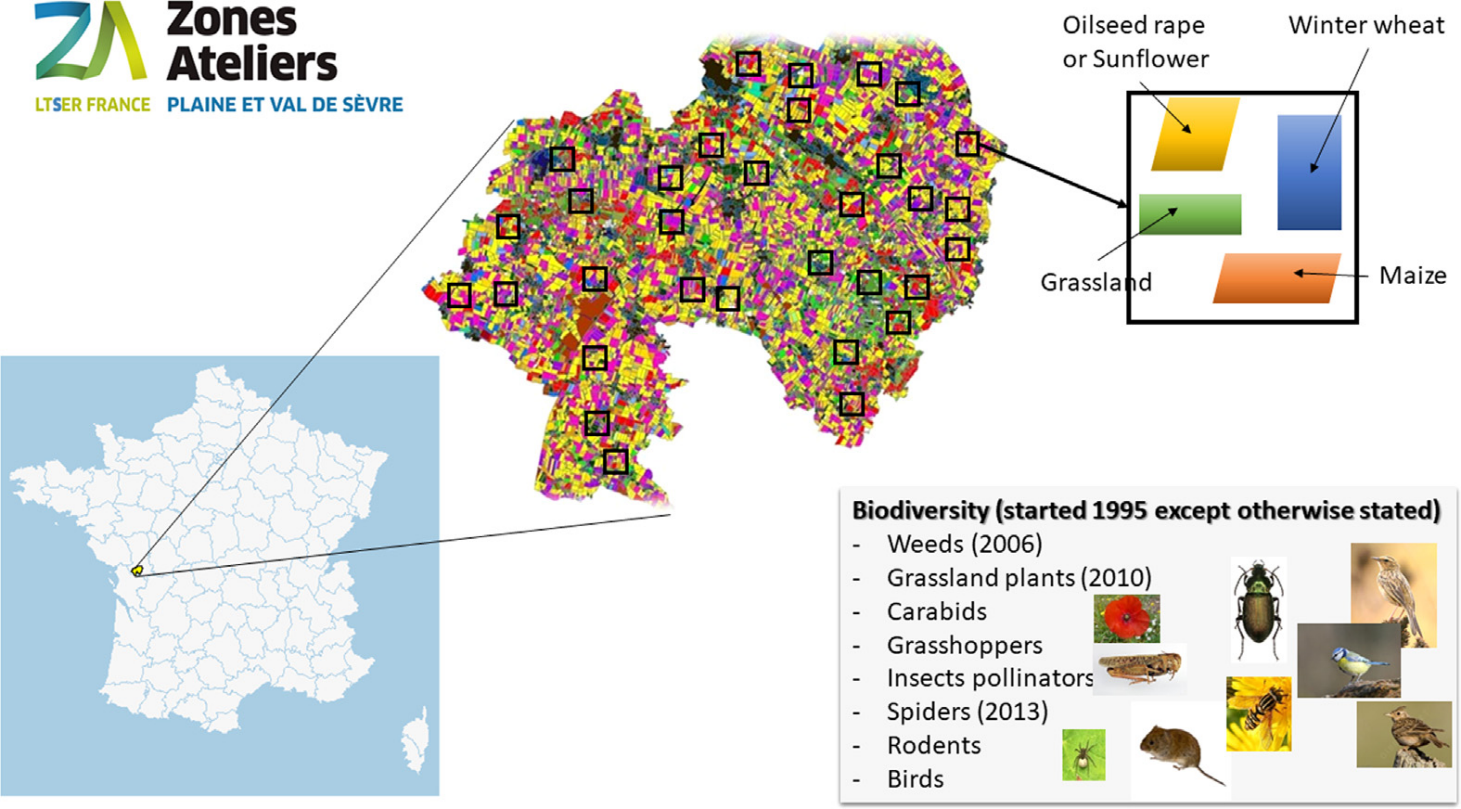
Description of the long term monitoring strategy in the LTSER platform Zone Atelier “Plaine & Val de Sèvre”. The LTSER platform is situated Center-West of France, South of Niort (yellow region on the France map). Colors on the LTSER map indicate the land use with among others winter cereal in yellow, maize in magenta, and grassland in green. Each year, 40 to 60 1-km² window (represented by the black squares) are selected and biodiversity is sampled in four fields with different land cover as presented on the top right part of the figure. The taxa that are monitored are presented at the bottom right of the figure with in brackets the year of the beginning of monitoring.

Since 2002, the spatial coordinates of the center of all sampling points (quadrat, line, point or transect) have been recorded using the Global Positioning System (GPS) with a precision of ~10 m. Prior to 2002, sampling locations were mapped as accurately as possible, and coordinates were extracted from GIS. Since 2010, 40 to 60 1-km² windows have been selected each year along landscape gradients (e.g. % of organic farming or diversity of crop types) that were chosen for particular research aims (Fig. 1). The windows are selected using available land cover maps (crops, semi-natural habitats, forests and built up areas) to create statistically independent landscape gradients [2] for testing hypothesis relating biodiversity and ecosystem functions to local farming intensity and landscape heterogeneity. The independent gradients reflect variations in composition (crop diversity) and configuration [3], as well as meadows, woodland and organic farming cover within the landscape 1-km² window. Landscape windows are selected using an algorithm written in R that moves a window over the whole of the ZA PVS, iteratively selecting the windows to minimize inter-gradient correlations. Biodiversity is surveyed in three to four fields in each of the 1-km² windows (Fig. 1), usually three arable fields and one meadow. Arable fields are most often cultivated with winter wheat, maize, oilseed rape or sunflower; however, when these main crops are not present in the window we also select spring pea or flax crop fields.

## Appendix A Supplementary material

.

Supplementary material **Table 1**. Overview of the protocols for the various taxa monitored in the LTSER Zone Atelier “Plaine & Val de Sèvre”.
