# Supplementary material for: Description of long-term monitoring of farmland biodiversity in a LTSER
Source: Data Brief. 2018 May 19;19:1310–3. doi: 10.1016/j.dib.2018.05.028 (PMC6139370; doi:10.1016/j.dib.2018.05.028)
Supplement: Supplementary file 1 — Supplementary material [file mmc1.docx]

**AUTHOR DECLARATION TEMPLATE**

I wish to confirm that there are no known conflicts of interest associated with this publication and there has been no significant financial support for this work that could have influenced its outcome.

Signed by the corresponding author on behalf of the authors:

Vincent Bretagnolle
