# Supplementary material for: Description of long-term monitoring of farmland biodiversity in a LTSER
Source: Data Brief. 2018 May 19;19:1310–3. doi: 10.1016/j.dib.2018.05.028 (PMC6139370; doi:10.1016/j.dib.2018.05.028)
Supplement: Supplementary file 2 — Supplementary material [file mmc2.docx]

***Supplementary material***

**Long-term monitoring protocols of biodiversity in farmland**

**Authors**: Vincent Bretagnolle^1,2^, Elsa Berthet^3^, Nicolas Gross^1,5^, Bertrand Gauffre^1,5^, Christine Plumejeaud^4^, Sylvie Houte^1^, Isabelle Badenhausser^1,5^, Karine Monceau^1^, Fabrice Allier^6,7^, Pascal Monestiez^1,5,8^ and Sabrina Gaba^1,2,9,10^

**Affiliations**:

^1^ CEBC, UMR 7372, CNRS & Université de la Rochelle, 79360 Villiers-en-Bois, France.

^2^ LTSER “Zone Atelier Plaine & Val de Sèvre”, CNRS, 79360 Villiers-en-Bois, France.

^3^ UMR SADAPT, INRA, AgroParisTech, Université Paris-Saclay, 16 rue Claude Bernard, 75005 Paris, France.

^4^ UMR LIENSs 7266 Université de la Rochelle et CNRS, 2 rue Olympe de Gouges, 17000 La Rochelle, France.

^5^ USC 1339, Centre d’Etudes Biologiques de Chizé, INRA, Villiers en Bois, 79360 Beauvoir sur Niort, France.

^6^ ITSAP-Institut de l’Abeille, Domaine Saint-Paul, CS 40509, 84914 Avignon, France.

^7^ UMT PrADE, CS 40509, 84914 Avignon, France.

^8^ BioSP, INRA, 84914 Avignon, France.

^9^ Agroécologie, AgroSup, INRA, Université de Bourgogne, 21065 Dijon, France.

^10^ USC 1339 INRA, Centre d’Etudes Biologiques de Chizé, 79360 Villiers-en-Bois, France.

**Contact email**: [vincent.bretagnolle@cebc.cnrs.fr](mailto:vincent.bretagnolle@cebc.cnrs.fr)

**Plants (crops)** – Since 2005, weeds have been recorded between March and June in about 100 to 250 fields (including at least 50-100 winter cereal fields) per year, in three selected fields in each 1-km² window (including mostly alfalfa, barley, oilseed rape, spring pea, sunflower, wheat). Three survey designs have been used successively in the selected arable fields. From 2006 to 2009, 32 4-m² quadrats on transects forming an eight-pointed star in the center of the field were surveyed (Fig. S1a). Each arm had four 4m2 plots located at 4, 12, 38 and 60m from the centre of the star. From 2009 to 2013, 10 quadrats of 4 m² on one transect in the center of the field and, in 2013, 40 quadrats of 1 m² on two parallel transects in the center of the fields were surveyed; field margins were also surveyed along a 50 m transect (Fig. S1b). The occurrence and abundance (using a semi-quantitative log scale) of individual weed species were recorded for each quadrat.

Since 2014, plants have been recorded for 80 0.25-m² quadrats on two parallel transects in the center of the fields and 20 0.25-m² quadrats in the field margin (Figure S1c). The size of the quadrat has been selected to ensure for the best representation of the plant community in the field while minimizing the survey time. Each 1m² quadrat is sub-divided into four sub-quadrats (Fig. S1). Species abundance is recorded using a presence/absence scale in each sub-quadrat. Plant phenology has also been recorded since 2016 (fruiting/flowering/green). All plants are identified and named according to [1-3], except for a few taxa for which the small seedling size and the absence of reproductive parts restricted the identification to genus level. Overall, the dataset currently contains about 2500 field surveys, half of which were for winter cereals, then alfalfa and then the other main crops (oilseed rape, maize, sunflower, etc.).


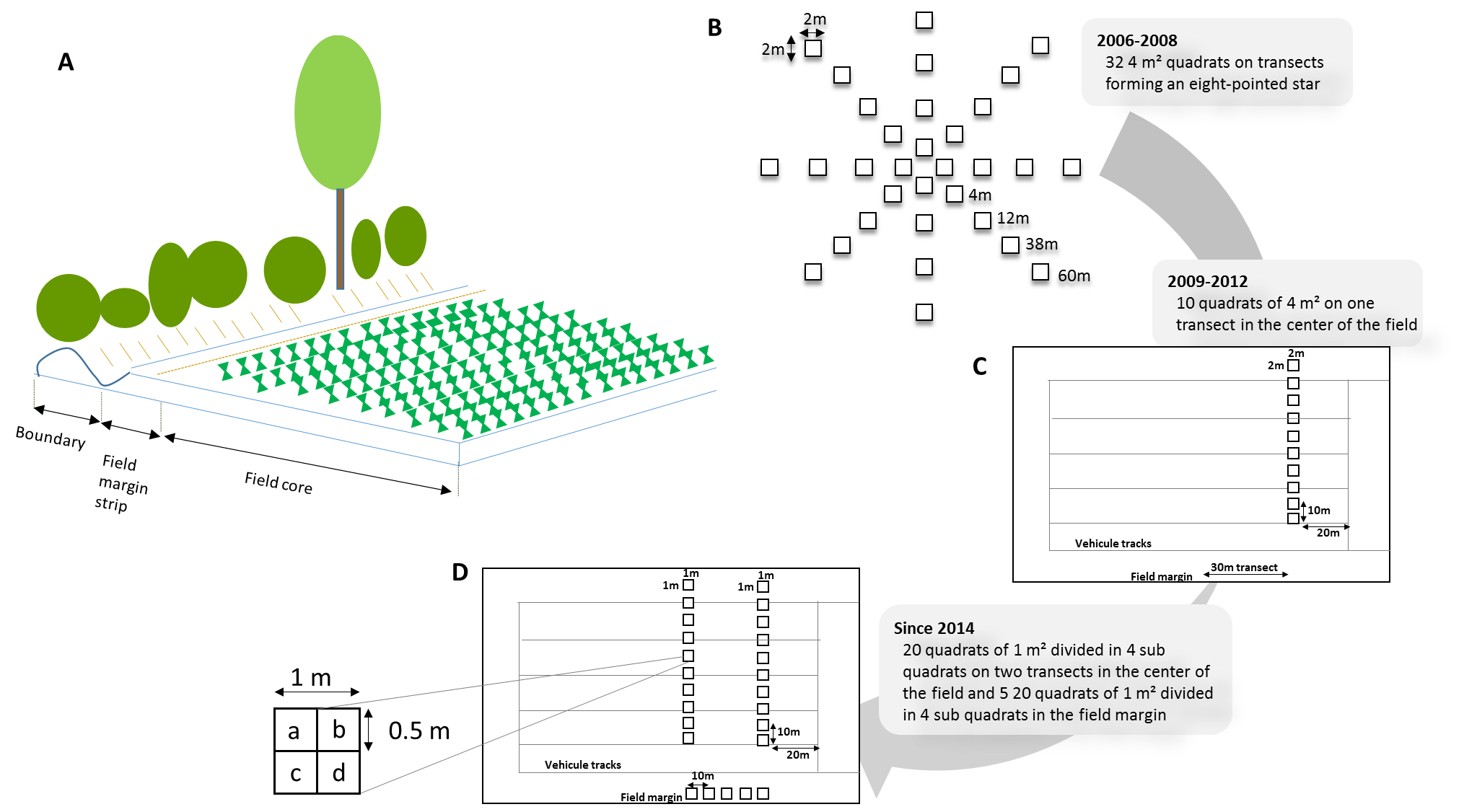


***Figure S1***: Description of the plant survey design in an arable field. A represents an arable fields with the different compartments. Two field compartments are surveyed: the field margin which is strip between the field boundary and the first crop row, and the field core. B-D show the different survey protocols that have been applied sin 2006. See main text for a detail description.

**Plants (meadows)** – The diversity and the abundance of plants in meadows have been monitored since 2011. In summer 2011, an initial survey was conducted by randomly selecting 209 meadows across the ZA PVS in order to determine the baseline for grassland plant diversity in the ZA PVS. 10 quadrats of 1 m² were located randomly within each field to estimate plant diversity and abundance. In each quadrat, the number of species was recorded and the percentage cover was estimated visually for each species within each quadrat. Relative abundance for each plant species was then calculated as the sum of the cover for the species in the 10 quadrats divided by the sum of the cover for all species. Meadow plant diversity has monitored regularly since 2013. Each survey includes 60 to 90 meadows following the protocol described above. Since 2015, we have applied the weed sampling protocol to meadow plants i.e. 20 sub-quadrats in the field margin (defined as in arable crops; Fig. S1) and 80 sub-quadrats along two parallel transects in the center of the field. The meadow protocol differs from the weed sampling protocol by cover estimates in one sub-quadrats of each quadrat in the field margin (five sub-quadrats) and in the center (twenty sub-quadrats) of the field. We also adjusted the size of the quadrats to reduce the survey time, i.e. grassland plants are surveyed in 0.5m² quadrats divided in four 0.25 x 0.25m² sub quadrats.

**Ground dwelling arthropods (carabid beetles and spiders)** – The activity-density of ground dwelling arthropods has been assessed using pitfall traps since 1994. This survey primarily targeted the carabid beetle community, but was eventually broadened to all invertebrates captured. From 1995 to 2012, three pitfall traps were placed between 10 m and 30 m from the field margin and 10 m from each other. Pitfall traps are particularly relevant to survey ground-dwelling arthropods such as carabid beetles [4]. Pitfall traps consists in plastic cups (8.5 cm in diameter and 7 cm deep), buried at the ground level, and filled with a solution to improve insect preservation. From 2009 to 2012, one additional trap was placed in the grassy field margin. These traps were filled with a 50% preservative solution of ethylene glycol (1995 to 2010), monopropylene glycol (2009 and 2010), ethanol (2011 to 2012) and since 2013 onwards, containing ten drops of odorless soap and 10g of salt per liter of water. The solution for the pitfall traps was changed to be harmless. The different preservative solutions used affected the measurement of carabid beetle activity-density but the differences between crops were not affected by this bias [5]. There were two to nine sampling campaigns each year, which were carried out year round, though mainly during the spring period (April-June). Pitfall traps were left in place for five (1995-2010) or four (2011-onward) effective days and, for a given year, set up at the same location for all campaigns. Since 2013, the two traps have been set in the margin (first crop row) and three others at 10 and 25m from the edge. The three fields selected in each 1-km² window are sampled twice a year from April to July (including alfalfa, meadows, oilseed rape, sunflower, maize and wheat). Arthropods are then stored in 70% ethanol and later identified to species level.

**Grasshoppers** – Surveys began in 2000 and have been intensified since 2003 in temporary and permanent grasslands as well as in artificial meadows such as alfalfa and clover. The sampling method was based on a 1 m² quadrat device which catches grasshoppers and allows them to be identified and counted inside the cage [6,7]. The device is thrown haphazardly 10 or 15 times in a meadow to achieve a precision of 20% to 30% for mean grasshopper density. Every year since 2003, a weekly survey of ten meadows from egg hatching in May to adult death in October was carried out to estimate the hatching period, juvenile and adult peaks in the season and the duration of the reproductive period. Between 40 and 200 meadows have been surveyed since 2000 at adult peak, in late July or early August depending on meteorological conditions, to estimate grasshopper densities at the landscape scale. Each year, fields have been randomly selected from all types of meadows found in the study site. Some additional sampling designs were set up for more specific aims such as understanding the colonization process or assessing the effects of landscape composition, landscape configuration and agricultural practices on alfalfa, on agri-environmental scheme and on sown grass margin strips. Most of grasshopper species may be easily identified at the species level using [8] except some *Chorthippus* and *Tetrix* species.

**Bees and hoverflies** – Bees and hoverflies have been surveyed since 2010 using field survey methods inspired by [9] and described by [10,11]. In addition to field surveys, an experimental managed honey bee population (50 hives) is monitored annually using standardized surveys of population dynamics using the ECOBEE long-term monitoring platform set up in 2008 [12]. The wild bee survey was carried out in two successive steps using two different methods: standardized sweep net captures of bees visiting flowers and colored pan traps. Sweep net captures of bees visiting flowers can be used to determine the bee / plant species interactions and provide better detection of honey bees and bumble bees than pan traps [9].

Since 2013, pollinators have been sampled along transects in 120-240 fields each year, using sweep nets [10]. The transects were 50 m in length were walked for 10 minutes, measured with a chronometer to ensure equal sampling effort, between 8:30 and 17:30 when the air temperature was above 15°C and the weather was sunny [13]. Coloured pan traps consisted in bowls of 12cm diameter, 10cm deep plastic sprayed fluorescent yellow (RAL 1026, Euro industry Supply, Stuttgart, Germany), sprayed fluorescent blue (Sparvar 3107, Euro industry Supply, Stuttgart, Germany) or left white. Different colours capture different pollinators by their colour preferences [9]. The traps were mounted on wooden stakes, with the height of the bowls being adjusted that they were at the vegetation canopy [9]. The bowls were filled with about 600 ml of water with drops of soap to catch insects. For a given field, pan traps were settled only once, left for 4 days and removed afterwards. Across all fields, pan traps were installed all-over spring (from April to June). Pan traps tend to undersample some taxonomic groups, e.g. bumble bees (genus *Bombus*) and colletid bees (genus *Colletes*) [9, 14]. The insects were then stored in 70 % ethanol and later identified to species level.

Since 2013, pan traps have been routinely set up in 180 to 240 fields with 12 traps per field, reduced to 6 since 2015. . The fields are selected from the 40 to 60 1-km² windows (thus being spatially extensive) to cover a wide range of environmental configurations.

**Butterflies** – Butterfly surveys started in 2013, and have been conducted along two 5 m wide 50 m long transects, along the field edge and inside the field. Surveys are mostly conducted on calm (Beaufort scale < 3), sunny days, when the temperature is above 15°C. The observer records all butterfly species observed within an imaginary 5 m cube (2.5 m to each side, 5 m in front and 5 m high) for approximately 10 min per transect [15]. Individuals that cannot be identified by sight are captured with a butterfly net for closer examination.

**Small Mammals -** Small mammals have been surveyed since 1995 onwards using trap lines. Sampling was restricted to April and June until 1999, after which there were occasional additional trapping campaigns in other months. From 1995 to 2011 the protocol was adapted from a standardized protocol [16] and used a 100 m trap line with 51 single capture live animal traps spaced every two meters, set for 24 hours. The fields sampled were selected in order to represent each major crop type while uniformly covering the whole study site. The site was subdivided in nine sectors of similar size. For a given sector and trapping session, 10 fields were selected to provide a uniform stratification between crop types (2-3 winter cereals, 1-2 oilseed rape, 1-2 alfalfa, 1-3 meadows, 1-2 spring sowed crops and 0-1 less common crops) along two transects crossing the sector (the June transect was orthogonal to the April transect). In 2011, the protocol was modified to maximize survival and welfare of the trapped animals and comply with the animal welfare policies that were introduced in France in 2013. In order to maintain the continuity of the time series, the same traps were used, but a second chamber was added to each trap (a plastic box) with a stock of wood shavings and food (wheat and carrot) [17]. Since 2013, each field has a 50-m trap line with 20 single capture live traps with feed boxes within the field and 5 traps in an orthogonal trap line in the field border. Two to three fields are selected within each 1-km² window and are sampled twice during the year in April and June. All animals are sexed, weighed and check for reproductive status, and then are released at the trapping site. The few dead animals (usually <5%) are collected and stored at -20°C for subsequent autopsies. Overall, we have trapped more than 6000 fields for about 300,000 trap-days over the whole study period. The most abundant species is the common vole. Two other species, the wood mouse and the greater white-toothed shrew account for about 30% and 20% of the catches, respectively. Since 2006, a small piece of ear is taken from common voles for genetic analysis.

**Birds** – Bird monitoring started in 1995 and three main approaches have been used depending on the taxon: nest searches and exhaustive counts in threatened flagship species (those that are targeted by NATURA 2000), nocturnal point counts for owls and stone curlews and diurnal point counts for passerines and other birds.

***Nest searches and exhaustive counts of threatened flagship species*** – The little bustard *Tetrax tetrax* has an exploded lek mating system in which males display in aggregated sites that females attend only for the purpose of mating [18]. In our study site, little bustards are migratory [19]. Males arrive on the breeding grounds from late March to early May. Since 1995, intensive searches for little bustards have been carried out on favorable plots throughout the study site during the breeding season, from late March to mid-July, by a team of 3 to 5 people, supported by other fieldworkers surveying other species in the study site. Little bustards are surveyed using vehicle-based searches exclusively, along all roads and tracks in the study site [20]. In addition little Bustard males have been individually identified based on breeding plumage [21,22], so that males that had moved from one place to another could be detected and not counted as new males. Females and fledglings, despite their highly cryptic behavior, are also intensively surveyed throughout the breeding season in order to provide a conservative estimate of their number in the study site [20]. This is especially possible during the post-breeding period, when little bustards gather in post nuptial groups, presumably in order to prepare for migration [23]. Montagu’s, hen and marsh harriers (*Circus* spp.) are similarly surveyed by nest searching. The nest searching effort is intensive and regular over the study period and site, starting in 1994, with an almost exhaustive census of breeding pairs each year [24]. Nests are visited at least 3 times during the breeding season, starting as early as possible after laying, in order to collect data on clutch size, number of nestlings and fledglings. Counting stone curlews *Burhinus oedicnemus* is not easy, due to the shyness of birds, their extended breeding season, and the variety of crops and habitats they use. In order to monitor the population size on our study site, we developed a standardized counting method that used limited resources so that it could be carried out on more than half of the study site by no more than five observers for about one week of field work. The method is based on the fact that about 80% of nests were in spring sown crops, that these crops (sunflower and maize) are less than 5-10 cm tall early in spring when about half of stone curlews have laid (and were therefore easy to find in the bare ground). Therefore every year since 2003, at the beginning of May, the stone curlews are counted during the daytime in the various sub-sectors of the study site.

***Passerine population*** – Passerine populations have been surveyed during the breeding season since 1995, using various survey designs. From 1995 onwards, a network of 160 fixed count points [25] was used. Initially, only a few species were surveyed but since 2006, the entire bird community has been surveyed. The sampling was stratified into eight sectors to ensure uniform coverage of the study site. Count points were distributed along two intersecting transects per sector, each transect with ten count points about 500m apart [26]. Observation radius was restricted to 200m from the observer to reduce any bias in detectability and avoid overlap of observations between two neighboring points. All birds observed, as well as their behavior (singing or not, flying or on the ground) were recorded on a field map. Counts lasted 10 min per sampling point from 1995 to 2000, a duration that was reduced to 5 min from 2001 onwards with negligible loss of probability of presence of at least skylarks and corn buntings [26,27]. Counts were performed in the morning (from 7:00 to 11:00 am), once in the breeding season until 2005 and twice since 2006.

The protocol was extended spatially in 2009 cover almost every 1 km² square of the study site. This larger spatial scale monitoring scheme was based on 355 point counts in 2009 and 2010, 260 points in 2011 and 284 points in 2012, about 750m apart on average and sampled twice during the breeding season (from early May to mid-June). All birds observed or heard within a 200 m radius in a 5 min period, and their behavior (singing, flying, foraging), were recorded on a field map [26].

Since 2013, a third, more robust, design with 260 count points has been surveyed twice a year, for 10 minutes (5 x 2-minute sessions) in order to increase the detection probability. Each year since, the 260 points are randomly resampled.

***Nocturnal birds*** – Since 1999, nocturnal surveys have been carried out for little owl *Athene noctua*, scops owl *Otus scops* and stone curlew, using the playback method (for owls) twice during the breeding season. Listening points were chosen to cover all urban areas, hedges and isolated trees. Even though the playback method is highly effective, resulting in an estimated 80-90% response rate, there are still individuals who do not respond to the playback [28]. Nocturnal playback surveys were first conducted in 1999 and continued biannually during the breeding season. Predetermined listening points have been established in the 24 communes of the ZA PVS and have been strictly maintained throughout the research study. Each point is visited in campaigns in April and June each year. Listening points are located about 500-750 meters apart along transects in each commune. After the broadcast, the observers mark the position of the birds on a map as precisely as possible. The presence of stone curlews is also recorded during these campaigns although stone curlew calls are not broadcast. The monitoring period starts one hour after sunset and finished three hours later. The listening time is 5 minutes for each point.

**References**

1. Hanf, M., Ackerunkräuter Europas mit ihren Keimlingen und Samen. 1982, Speyer: Klambt-Druck.
2. Jauzein, P., **Flore des champs cultivés**. 1995: Editions Quae.
3. Marmarot, J., R. Psaiski, and R. Rouquier, **Mauvaises herbes des cultures**. 1997, Paris: ACTA.
4. Luff, M.L., **Some features influencing the efficiency of pitfall traps**. Oecologia, 1975. 19: 345.
5. Marrec, R., et al., **Crop succession and habitat preferences drive the distribution and abundance of carabid beetles in an agricultural landscape**. Agriculture, Ecosystems & Environment, 2015. 199: p. 282-289.
6. Badenhausser, I., et al., **Acridid (Orthoptera: Acrididae) abundance in Western European Grasslands: sampling methodology and temporal fluctuations**. Journal of Applied Entomology, 2009. 133(9‐10): p. 720-732.
7. Badenhausser, I., P. Amouroux, and V. Bretagnolle, **Estimating acridid densities in grassland habitats: a comparison between presence-absence and abundance sampling designs**. Environmental entomology, 2014. 36(6): p. 1494-1503.
8. Defaut, B., Biometrie des types des Caeliferes de France (Orthoptera).**1. Definition des parametres mesurees. 2. Mensurations chez les Tridactylidae, Tetrigidae, Pyrgomorphidae, Pamphagidae et Acrididae Calliptaminae**. Materiaux Orthopterique & Entomocenotique, 2012. 17, 21–56.
9. Westphal, C., et al., **Measuring bee diversity in different European habitats and biogeographical regions.** Ecological Monographs, 2008. 78(4): p. 653-671.
10. Rollin, O., et al., **Differences of floral resource use between honey bees and wild bees in an intensive farming system**. Agriculture, Ecosystems & Environment, 2013. 179: p. 78-86.
11. Rollin, O., et al., **Habitat, spatial and temporal drivers of diversity patterns in a wild bee assemblage**. Biodiversity and Conservation, 2015. 24(5): p. 1195-1214.
12. Odoux, J.-F., et al., ECOBEE: **a tool for long-term honey bee colony monitoring at the landscape scale in West European intensive agroecosystems**. Journal of Apicultural Research, 2014. 53(1): p. 57-66.
13. Perrot, T., et al., **Experimental evidence in real field conditions shows that bees can increase Oilseed Rape yield up to 30%**. in review.
14. Roulston, T.a.H., S.A. Smith, and A.L. Brewster, **A comparison of pan trap and intensive net sampling techniques for documenting a bee (Hymenoptera: Apiformes) fauna**. Journal of the Kansas Entomological Society, 2007. 80(2): p. 179-181.
15. Pollard, E. and T.J. Yates, **Monitoring butterflies for ecology and conservation: the British butterfly monitoring scheme**. 1994: Springer Science & Business Media.
16. Spitz, F., et al., **Standardisation des piégeages en ligne pour quelques espèces de rongeurs**. 1974.
17. Le Quilliec, P. and S. Croci, **Piégeage de micromammifères: une nouvelle boîte-dortoir pour le piège non vulnérant INRA**. Le cahier des Techniques INRA Numéro Spécial sur les Méthodes et outils pour l’observation et l’évaluation des milieux forestiers, prairiaux et aquatiques, 2006.
18. Jiguet, F., B. Arroyo, and V. Bretagnolle, **Lek mating systems: a case study in the Little Bustard *Tetrax tetrax*.** Behavioural Processes, 2000. 51(1): p. 63-82.
19. Villers, A., et al., **Migration of wild and captive‐bred Little Bustards *Tetrax tetrax*: releasing birds from Spain threatens attempts to conserve declining French populations**. Ibis, 2010. 152(2): p. 254-261.
20. Bretagnolle, V., et al., **Rapid recovery of a depleted population of Little Bustards *Tetrax tetrax* following provision of alfalfa through an agri‐environment scheme**. Ibis, 2011. 153(1): p. 4-13.
21. Arroyo, B. and V. Bretagnolle, **Field identification of individual Little Bustard *Tetrax tetrax* males using plumage patterns**. Ardeola, 1999. 46(1): p. 53-60.
22. Jiguet, F. and D. Ollivier, **Male phenotypic repeatability in the threatened Little Bustard Tetrax tetrax: a tool to estimate turnover and dispersal**. Ardea, 2002. 90(1): p. 43-50.
23. Casas, F., et al., **Effects of hunting on the behaviour and spatial distribution of farmland birds: importance of hunting‐free refuges in agricultural areas**. Animal Conservation, 2009. 12(4): p. 346-354.
24. Millon, A., et al., **Comparative breeding biology of Hen Harrier and Montagu’s Harrier: an 8‐year study in north‐eastern France.** Ibis, 2002. 144(1): p. 94-105.
25. Bibby, C.J., Bird census techniques. 2000: Elsevier.
26. Brodier, S., et al., **Local improvement of skylark and corn bunting population trends on intensive arable landscape: a case study of the conservation tool Natura 2000. Animal conservation**, 2014. 17(3): p. 204-216.
27. Bonthoux, S. and G. Balent, **Point count duration: five minutes are usually sufficient to model the distribution of bird species and to study the structure of communities for a French landscape**. Journal of Ornithology, 2012. 153(2): p. 491-504.
28. Génot, J.-C., **La chevêche d'athéna, *Athene noctua*, dans la Réserve de la biosphère des Vosges du Nord: de 1984 à 2004.** 2005.
